# Validation of the Lithuanian Version of the Walking Impairment Questionnaire in Patients with Peripheral Arterial Disease

**DOI:** 10.3390/medicina60010147

**Published:** 2024-01-13

**Authors:** Tomas Baltrūnas, Karolis Medelis, Augustė Melaikaitė, Austėja Račytė, Gabija Pikturnaitė, Rokas Baltušnikas, Ieva Laucevičienė, Kęstutis Ručinskas

**Affiliations:** 1 Faculty of Medicine, Vilnius University, 03101 Vilnius, Lithuania; auguste.melaikaite@mf.stud.vu.lt (A.M.); austeja.racyte@mf.stud.vu.lt (A.R.); gabija.pikturnaite@mf.stud.vu.lt (G.P.); ieva.lauceviciene@santa.lt (I.L.); kestutis.rucinskas@santa.lt (K.R.); 2 Vilnius University Hospital Santaros Clinics, 08661 Vilnius, Lithuania; karolis.medelis@santa.lt; 3 Emergency Department, Republican Vilnius University Hospital, 04130 Vilnius, Lithuania; rokas.baltusnikas@gmail.com

**Keywords:** walking impairment questionnaire, peripheral arterial disease, intermittent claudication, validation studies

## Abstract

*Background and Objectives*: The Walking Impairment Questionnaire (WIQ) is a short and simple tool to measure walking impairment for patients with peripheral arterial disease requiring no special equipment or trained staff. The aim of this study was to assess the validity and reliability of the culturally adapted Lithuanian WIQ version in patients with intermittent claudication. *Materials and Methods*: In total, 40 patients with intermittent claudication and ankle–brachial index < 0.90 participated in this study. Reliability and internal consistency of the questionnaire were assessed by the intra-class correlation coefficient (ICC) and Cronbach’s alpha (α), respectively. Validity was determined by correlations between the WIQ scores and a subjective test (Quality of Life 5 Dimension Questionnaire 3 Level Version (EQ-5D-3L)) and objective tests (6 min walk test (6MWT), treadmill test, and ankle–brachial index). *Results*: The test–retest reliability was assessed as excellent with an intraclass correlation coefficient of 0.90. The values of Cronbach’s alpha were 0.957 (I time) and 0.948 (II time) and indicated an excellent internal consistency. Statistically significant Spearman correlations were detected between the WIQ and walking distances on the 6MWT (rho 0.514, *p* < 0.001) and treadmill test (rho 0.515, *p* < 0.001). Higher WIQ scores were associated with longer walking distances and duration. Moderate negative and low negative correlations were found between the WIQ and EQ-5D-3L scores. *Conclusions*: The Lithuanian version of culturally adapted WIQ demonstrates reliability and validity for patients with intermittent claudication, supported by two different walking tests showing statistically significant moderate Spearman correlations.

## 1. Introduction

Peripheral arterial disease (PAD) includes conditions that cause the obstruction of arterial blood flow, excluding the coronary and intracranial vessels, when the ankle–brachial index is lower than 0.90 [1,2]. According to systematic reviews, the prevalence of PAD was approximately 202 million individuals in 2010, and it increased to an estimated 237 million people in 2015 [3,4]. Peripheral arterial disease is associated with functional impairment, while intermittent claudication (IC) is the first and the most frequent symptom of PAD [1]. IC is classically defined as pain, including one or both of the lower extremities, that starts on walking and requires a stop to relieve pain [5]. Based on several different studies, the prevalence of IC within a general population ranges from 0.7% to 7% [6,7,8,9,10]. Additionally, McDermott et al. conducted a study involving patients with an ABI < 0.9 and found that 32% of individuals diagnosed with PAD exhibited typical IC symptoms [11].

The treadmill test is considered a standard method to objectively assess walking impairment for PAD patients [12]. However, treadmill testing requires specific equipment, trained staff, is time-consuming, and is inappropriate to use in primary care settings or epidemiological studies. Moreover, the treadmill test does not detect walking impairment occurring in daily life precisely, and individuals may encounter challenges walking on a treadmill due to balance disorders, frailty, or walking limitations unrelated to PAD [2]. While the majority of IC patients receive outpatient treatment, the WIQ would be a more accessible tool for assessing the severity of walking impairment. 

The Walking Impairment Questionnaire (WIQ) is a short and simple questionnaire measuring ability to walk in three different subscales: walking distance, walking speed, and stair climbing [13]. The WIQ has been validated in many countries, including the USA, the Netherlands, Brazil, Spain, China, South Korea, and others [13,14,15,16,17,18]. The original WIQ version was translated into the Lithuanian language and adjusted for the metric system in a previous study [19]. The present study aimed to determine if the Lithuanian version of the culturally adapted WIQ is reliable and valid for PAD patients with intermittent claudication.

## 2. Materials and Methods

### 2.1. Participants and Study Design

A prospective study including 40 PAD patients with IC was performed. Respondents were selected from Vilnius University Hospital Santaros Clinics from 2020 February to 2023 June. The inclusion criteria were PAD with IC (grade I, categories 1–3, according to Rutherford classification [20]), ankle–brachial index (ABI) < 0.9, and age between 50 and 90. Exclusion criteria were inability to walk due to orthopedical, neurological, or other comorbidities, dependency on oxygen, and insufficient knowledge of the Lithuanian language. Baseline data, presence of comorbidities (end-stage renal disease, diabetes mellitus, previous stroke, previous myocardial infarction, hypertension), and use of statins were collected. Informed consent was obtained from every participant. This study was approved by the Vilnius Regional Bioethics Committee on 2020 February 25 Nr. 2020/2-1198-684.

### 2.2. Walking Impairment Questionnaire (WIQ)

The WIQ is a short, easy-to-fill-out questionnaire that was developed by Regensteiner et al. in 1990 for patients with IC to determine the severity of walking impairment in patients with PAD [21]. It contains three subscales: walking distance, walking speed, and ability to climb stairs [13]. 

All subscales of the WIQ were estimated by a Likert scale [22], where 0 represents inability to walk, while 4 indicates no difficulties in walking. The subscale of walking distance measures ability to walk different distances from walking indoors (around the house) to 1000 m. The walking speed subscale indicates ability to walk at different speeds from slowly walking to running or jogging. The stair climbing subscale determines ability to climb from 1 to 3 flights of stairs.

Questionnaire scoring was based on a formula presented in the original version of the WIQ [21] and was double-checked with the Dutch version of the WIQ, which was culturally adapted from imperial (feet) to metric system units [14]. The walking distance subscale score was calculated by multiplying distance in meters and the Likert score of that distance. Then, the products of each distance were summed and divided by the maximum possible score of the distance subscale. For the walking speed subscale, different speeds were given weighting multipliers (from 1.5 to 5 miles per hour, as in the original version [21]) which were multiplied by the Likert score. The products were summed and divided by the maximum score of the speed subscale. The score of the climbing stairs subscale was assessed by multiplying the number of flights of stairs by the Likert score and divided by the maximum score of the climbing stairs subscale. The results of each subscale were multiplied by 100 to obtain scores in percentages. The total score of the WIQ was calculated as a mean of three subscale scores. 

The original English version of the WIQ was translated into the Lithuanian language and culturally adapted in a previous study [19]. The translation was made by two independent translators; translations were compared and then translated back into the English language to compare with the original version. 

### 2.3. European Quality of Life 5 Dimension Questionnaire 3 Level Version (EQ-5D-3L)

EQ-5D-3L is a short quality of life questionnaire containing five questions on five different domains (mobility, self-care, usual activities, pain/discomfort, and anxiety/depression) and the vertical visual analogue scale (VAS) [23]. Each question was assessed by the Likert scale, where 1 implies good quality of life, while 3 indicates difficulties in different situations. This scaling is opposite to the WIQ scaling system, assuming negative Spearman correlations between these two questionnaires. The vertical VAS was set from 0 to 100, where 0 means ‘the worst imaginable health state’ and 100 represents ‘the best imaginable health state’. Spearman correlations between the VAS and WIQ were expected to be positively correlated.

### 2.4. 6-Minute Walk Test (6MWT)

According to the latest guidelines from the European Society for Vascular Surgery, the 6 min walk test (6MWT) should be the primary test for assessing walking capacity and evaluating the severity of intermittent claudication in PAD patients [2]. This is considered more representative of everyday ambulatory function compared to treadmill testing. Moreover, the walking distance of the 6MWT closely correlates with walking capacity outdoors in patients with IC [24]. During the test, participants were asked to walk as far as possible in 6 min, and stopping for a rest when needed was permitted. The total distance walked in 6 min was recorded.

### 2.5. Treadmill Test

The treadmill test is a conventional test used to establish peripheral arterial disease diagnosis and to quantify severity [12]. In addition, the maximum walking distance measured during a graded treadmill test may be deemed the most trustworthy parameter for the treadmill [2]. In our study, the test started with a 0% incline and was increased by 2% every 2 min. The speed of the treadmill was constant and equal to 3.2 km/h. The test was terminated once the patient experienced IC. Walking duration, maximum walking distance, and reached incline angle were recorded.

### 2.6. Ankle–Brachial Index (ABI)

The ABI is a noninvasive and easily accessible method for diagnosing and estimating the severity of lower limb PAD [2]. The ABI is a ratio between the systolic blood pressures of the ankle and the upper extremity. An ABI lower than 0.9 indicates PAD with serious stenosis [25]. In our study, the ABI was measured with the Hokanson MD35 model (Bellevue, WA, USA) separately for both sides (left and right). The lower ABI was chosen for the analysis.

### 2.7. Validation Process

Reliability of the Lithuanian version of the WIQ was determined by internal consistency and test–retest reliability. Internal consistency determines the degree to which the test items jointly measure the same construct and was calculated in Cronbach’s alpha (α) [26]. Test–retest reliability was estimated by completing the WIQ twice and assessed by the intraclass correlation coefficient (ICC) [27]. The period between the first and second tests was two weeks. This duration was considered to be long enough for the patient to not remember their answers but short enough not to have a significant treatment effect.

The validity of the WIQ was measured by Spearman correlations with functional tests (6MWT and treadmill tests) and a subjective test (EQ-5D-3L). We have also assessed the Spearman correlations between the WIQ and ABI. Spearman correlations were assessed between each item and WIQ subscores, as well as the WIQ total score. In addition, we have conducted a power study.

### 2.8. Statistical Analysis

Demographic data and the results of walking tests, questionnaires, and the ABI were given as a mean and standard deviation (SD) for normal distribution based on the Shapiro–Wilk test [28]. Otherwise, the data were illustrated using a median and interquartile range (IQR). Comorbidities were presented as the number and the percentage of participants. 

The ICC was measured to determine test–retest reliability corresponding to ICC (3, B) by the conventions of Shrout and Fleiss [27]. The confidence interval was set to 95%. ICC < 0.50 was determined to be poor, 0.50 ≤ ICC < 0.75—moderate, 0.75 ≤ ICC < 0.90—good, and ICC ≥ 0.90—excellent [29]. Internal consistency was assessed by calculating Cronbach’s alpha(α). Based on George and Mallery’s rule of thumb, α ≥ 0.90 was determined as excellent, 0.80 ≤ α < 0.90—good, 0.70 ≤ α < 0.80—acceptable, 0.60 ≤ α < 0.70—questionable, 0.50 ≤ α < 0.60—poor, and <0.50—unacceptable internal consistency [30]. Correlations between the WIQ and 6MWT, treadmill test, ED-5D-3L, and ABI were assessed using Spearman correlation coefficients. Spearman correlations (Spearman’s rho) ≥ 0.90 were interpreted as very high, 0.70 ≤ rho < 0.90—high, 0.50 ≤ rho < 0.70—moderate, and 0.30 ≤ rho < 0.50—low, while correlations with rho < 0.30 were determined to be insignificant [31]. The statistical power analysis was conducted to assess the probability of rejecting the null hypothesis when it is false [32]. 

Statistical analysis was performed with JASP version 0.17.3 for Windows and G Power version 3.19.6. 

## 3. Results

### 3.1. Baseline Data

In total, 40 PAD patients with IC were enrolled in this study. One patient was excluded after not arriving for the functional (6MWT and Treadmill) tests. A total of 84.6% of the participants were men, and the mean age was 68.8 (SD 8.9). Regarding the occurrence of comorbidities, 57.5% of the participants had elevated arterial blood pressure, 35.0% were prescribed statins, 25.0% had a history of myocardial infarction, 20.0% were diagnosed with diabetes mellitus, 12.5% had a prior history of stroke, 12.5% were suffering from end-stage renal disease, and 10.0% were afflicted by chronic obstructive pulmonary disease.

The mean score of the WIQ total score was 48.8 (SD 20.8), and the mean ABI was 0.59 (SD 0.12). The median walking distances during the 6MWT and treadmill test were 390.0 m (IQR 108.0) and 80.0 m (IQR 60.0), respectively. The remaining results of the objective (6MWT, treadmill test) and subjective (EQ-5D-3L) tests are shown in Table 1.

### 3.2. Test–Retest Reliability and Internal Consistency

The ICC for the test–retest reliability for the total WIQ score was 0.90 (excellent). The ICCs for the walking distance, walking speed, and stair climbing subscales were 0.91 (excellent), 0.84 (good), and 0.80 (good), respectively (Table 2). The internal consistency, represented by Cronbach’s alpha (α), for the total WIQ score was assessed as excellent, with values equal to 0.96 (I time) and 0.95 (II time). Internal consistency values of each WIQ subscale are presented in Table 3.

### 3.3. Validity

**Figure 1:**
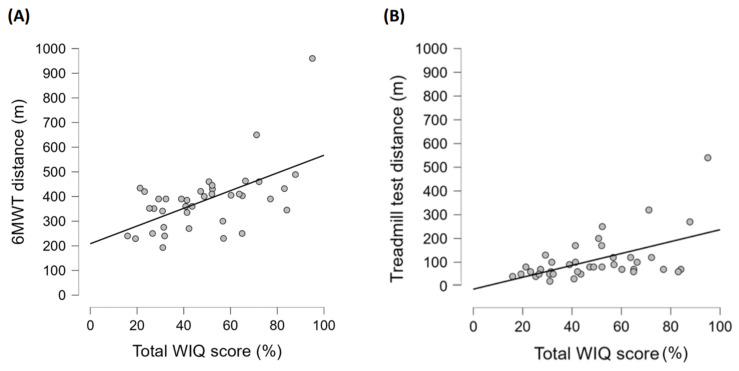
(**A**) The total WIQ score plotted against the distance of the 6MWT; (**B**) the total WIQ score plotted against the distance of the treadmill test.

Correlations between the WIQ and walking distance during two functional tests (6MWT and treadmill test) were moderate, with Spearman correlation coefficients of 0.51 and 0.52 (both *p* < 0.001) and power values of 0.93 and 0.94, respectively. Total WIQ scores were plotted against the distances of the 6MWT and treadmill test in Figure 1. Furthermore, higher WIQ scores were associated with longer walking distances and duration (Table 4). 

There was a low positive correlation between the total WIQ score and walking duration during the treadmill test (rho = 0.45, *p* = 0.004). However, no significant correlation was observed between the total WIQ score and the incline angle reached during the treadmill test.

Correlations between the WIQ and EQ-5D-3L (subjective test) were estimated to fall between moderate negative and low negative correlations. Spearman correlation coefficients between the total WIQ score and EQ-5D-3L domains representing anxiety/depression (rho = −0.61, *p* < 0.001), usual activities (rho = −0.51, *p* < 0.001), and mobility (rho = −0.48, *p* = 0.002) were higher than self-care (rho = −0.39, *p* = 0.014) and pain/discomfort (rho = −0.34, *p* = 0.032). A low positive correlation (rho = 0.49, *p* = 0.001) was observed between the WIQ and VAS. The remaining correlations between the WIQ and EQ-5D-3L are presented in Table 5. 

There were no significant correlations between the ABI and total WIQ score, walking speed subscale, or stair climbing subscale. A significant correlation was observed between the ABI and WIQ walking distance subscale, and this was determined as a low positive correlation (rho = 0.38, *p* = 0.018).

Power analysis was conducted due to the small sample size. When the Spearman correlation coefficient (rho) is 0.5, a sample size of 29 is deemed sufficient. In contrast, when the correlation coefficient is 0.3, a larger sample size of 84 is necessary. These calculations are made under the assumption of rejecting the null hypothesis, which posits that there is no correlation. Furthermore, these computations were performed with a target statistical power of 0.8, and α error was set at 0.05.

## 4. Discussion

The Lithuanian version of the WIQ showed good test–retest reliability, internal consistency, and correlations with functional tests (6MWT and treadmill). The Dutch version of the WIQ was the first version adapted for the metric system [14]. The main limitation of that study was that the treadmill test may not reflect the actual walking distance in daily life. Therefore, we included both the treadmill test and 6MWT, which illustrate daily life walking more closely [33]. Relationships between the WIQ and walking distances in two different tests (6MWT and Treadmill) were significant with moderate Spearman correlation coefficients. Finally, the positive correlation between walking distances and the WIQ, as illustrated by quartile scores, shows that the WIQ could help to differentiate patients by the severity of their walking impairment.

Despite the objective measuring of the ABI, no or low significant correlation between the ABI and WIQ was expected based on previous WIQ validation studies in other countries [13,17,34]. Furthermore, we found no significant correlations between the ABI and walking distances on functional tests (6MWT (rho = 0.23, *p* = 0.165) and the treadmill test (rho = 0.17, *p* = 0.303)). A recent meta-analysis of the toe–brachial index (TBI) and ankle–brachial index (ABI) in the diagnosis of PAD in lower extremities suggested a large heterogeneity in sensitivity for the ABI, better sensitivity of the TBI (82% vs. 52%), and better specificity of the ABI (77% vs. 94%) [35]. These observations show that the WIQ might be a more reliable tool to determine walking impairment severity in patients with PAD than a classical ABI test. We also indicate the importance of further studies to test the reliability of the ABI for determining the severity of walking impairment. 

The clinical significance of the WIQ validation has several aspects. Although it may not be the main diagnostic test for PAD with IC, the WIQ could evolve into a crucial tool for assessing the severity of walking impairment, as indicated by quartile scores. While the PAD is closely related to other cardiovascular diseases, such as coronary artery disease, carotid artery disease, and renal artery disease, the early determination of PAD severity could have a significant impact on outcomes. Moreover, the WIQ is a fast, easy to complete method requiring no specialized tools or trained staff. Finally, the validation of the Lithuanian version of the WIQ provides a good tool for further studies involving PAD patients.

There were several limitations of this study. Firstly, the sample size of this study was quite small and was not designed to represent all social backgrounds. In addition, not assessing the mental abilities of the participants may have affected the WIQ results of patients with dementia, post-stroke complications, or other cognitive dysfunction symptoms. Five questions of the EQ-5D-3L questionnaire might not give sufficient information about quality of life. As questionnaires give subjective results, a more comprehensive quality of life questionnaire (such as Medical Outcome Study Questionnaire Short Form 36 (SF-36) or Lifestyle and Clinical Survey) should be chosen [14,15,34]. The last limitation was the cultural adaptation of the WIQ. Standard size living blocks are not common in Lithuanian architecture, so questions in the WIQ distance subscale were only presented in meters, which could have made it difficult for the subjects to evaluate distances properly. Moreover, the lowest correlation observed between the WIQ stair climbing subscale and other tests could have been affected by the term ‘flight of stairs’, which is not widespread in the Lithuanian language. It would have been more accurate to add a number of how many stairs to each question of the stair climbing subscale, as occurred in the study of the validation of the Brazilian version of the WIQ [15]. 

Based on the lowest correlations between the WIQ stair climbing subscale and other results, further studies are needed to assess the relationship between the WIQ and functional test directly associated with stair climbing. Furthermore, a wide epidemiological study is necessary to determine whether the WIQ is capable of indicating the severity of walking impairment for PAD patients more precisely than the classical ABI test. Finally, based on the latest guidelines from the European Society for Vascular Surgery, further studies are needed to determine whether the WIQ is capable of differentiating PAD patients from outpatients, minimally invasive or open surgical treatment, as well as assessing the treatment of IC patients [2].

## 5. Conclusions

Reliability was assessed with good to excellent test–retest reliability and excellent internal consistency. The validation process involved the inclusion of two different walking tests, and statistically significant moderate correlations were observed with both assessments. Additionally, correlations between the WIQ and EQ-5D-3L were significant, ranging from moderate negative to low negative correlations. To conclude, the Lithuanian version of the culturally adapted WIQ is a reliable, valid, and clinically significant tool for quickly assessing the severity of walking impairment in PAD patients with IC without the need for trained staff and specialized expensive tools.

## Figures and Tables

**Table 1:** Baseline data.

Tests	Mean (±SD)	Median (±IQR)
WIQ total	48.8 (±20.8)	
WIQ distance		26.0 (±42.0)
WIQ speed	40.5 (±23.8)	
WIQ stair climbing		68.8 (±26.0)
6MWT distance		390.0 (±108.0)
Treadmill distance		80.0 (±60.0)
Treadmill duration		104.0 (±80.0)
Treadmill incline angle		0.0 (±2.0)
ABI	0.59 (±0.12)	
EQ-5D-3L Total		8.0 (±2.0)
EQ-5D-3L Mobility		2.0 (±1.0)
EQ-5D-3L Self-care		1.0 (±1.0)
EQ-5D-3L Usual activities		1.5 (±1.0)
EQ-5D-3L Pain/discomfort		2.0 (±0.0)
EQ-5D-3L Anxiety/depression		2.0 (±1.0)
VAS	55.0 (±14.1)	

**Table 2:** Test–retest reliability (ICC).

WIQ	Time I Mean (SD)	Time II Mean (SD)	Change of Scores Mean (SD)	ICC	95% Confidence Interval
Total	47.37 (21.82)	50.17 (20.76)	−2.80 (9.52)	0.90	0.82–0.95
Distance	34.09 (28.37)	33.65 (28.31)	0.43 (12.45)	0.91	0.83–0.95
Speed	38.57 (25.39)	42.50 (24.27)	−3.93 (13.94)	0.84	0.72–0.91
Stair climbing	69.44 (22.36)	74.36 (19.63)	−4.92 (13.48)	0.80	0.64–0.89

**Table 3:** Internal consistency (Cronbach’s alpha (α)).

WIQ	Time I Cronbach’s Alpha(α)	95% Confidence Interval (I)	Time II Cronbach’s Alpha(α)	95% Confidence Interval (II)
Total	0.96	0.93–0.97	0.95	0.92–0.97
Distance	0.95	0.92–0.97	0.94	0.91–0.97
Speed	0.93	0.88–0.96	0.89	0.82–0.94
Stair climbing	0.87	0.78–0.93	0.81	0.70–0.89

**Table 4:** Quartiles of the Lithuanian version of WIQ scores compared with walking distances.

WIQ Total Score in Quartiles	6MWT Distance (m)–Mean (SD)	Treadmill Distance (m)–Mean (SD)	Treadmill Duration (s)–Mean (SD)
0.0–31.2	320.0 (85.7)	59.0 (30.0)	88.4 (40.5)
31.2–47.2	342.6 (61.0)	79.0 (39.6)	120.2 (46.5)
47.2–64.4	387.8 (74.4)	131.1 (62.5)	165.9 (77.6)
64.4–100.0	484.2 (196.5)	168.0 (160.2)	223.0 (198.9)

**Table 5:** Spearman correlation coefficients between the WIQ and EQ-5D-3L.

	WIQ Total	WIQ Distance	WIQ Speed	WIQ Stair Climbing
EQ-5D-3L Total	−0.67 ***	−0.64 ***	−0.69 ***	−0.43 **
EQ-5D-3L Mobility	−0.48 **	−0.46 **	−0.43 **	−0.29
EQ-5D-3L Self-care	−0.39 *	−0.34 *	−0.52 ***	−0.22
EQ-5D-3L Usual activities	−0.51 ***	−0.47 **	−0.56 ***	−0.35 *
EQ-5D-3L Pain/discomfort	−0.34 *	−0.32 *	−0.30	−0.20
EQ-5D-3L Anxiety/depression	−0.61 ***	−0.59 ***	−0.57 ***	−0.41 **

* *p* < 0.05, ** *p* < 0.01, *** *p* < 0.001.

## Data Availability

The data presented in this study are available on request from the corresponding author.
